# Tissue microarray staining reveals PLD1 and Sp1 have a collaborative, pro-tumoral effect in patients with osteosarcomas

**DOI:** 10.18632/oncotarget.20605

**Published:** 2017-09-01

**Authors:** Xiao-Xu Wang, Ying Liao, Liang Hong, Zhi Zeng, Tang-Bo Yuan, Xue Xia, Jian Qin

**Affiliations:** ^1^ Department of Orthopaedics, The Second Affiliated Hospital of Nanhua University, Hengyang, Hunan, People’s Republic of China; ^2^ Department of Rehabilitation, The First Affiliated Hospital of Nanhua University, Hengyang, Hunan, People’s Republic of China; ^3^ Department of Orthopaedics, Sir Run Run Hospital, Nanjing Medical University, Nanjing, Jiangsu, China

**Keywords:** osteosarcoma, PLD1, Sp1, prognosis, immunohistochemistry

## Abstract

It has been reported that phospholipase D1 (PLD1) - a key enzyme involved in lipid metabolism - is important for the initiation and progression of various human solid cancers; however, its biological significance and regulation in human osteosarcomas remain elusive. In this study, We found that PLD1 and Specificity Protein 1 (Sp1) expression were elevated in 137 osteosarcoma specimens with immunohistochemical staining. Our results showed that both PLD1 and Sp1 were expressed at much higher rates in the cancerous tissue compared to adjacent normal tissue. A correlation analyze also indicated that PLD1 was significantly associated with lactate dehydrogenase expression (*p* = 0.041) and the Enneking stage (*p* = 0.000), while Sp1 was significantly associated with the nuclear grade (*p* = 0.024). Furthermore, survival analyses showed that elevated PLD1 confers a poor prognosis on patients with osteosarcomas, acting as an independent prognostic factor. Of note, we showed a positive correlation between PLD1 and Sp1 expression in the cancer tissues (*r* = 0.357; *p* < 0.001). High co-expression of the two molecules results in the worst prognosis for the patients, and can also be regarded as independent prognostic factor (*p* = 0.001; HR = 2.71; 95% CI 1.53–4.80).

## INTRODUCTION

As the most common primary malignant bone tumor in children and adolescents, osteosarcomas arise most frequently in the metaphysis of long bones [1]. The disease is highly aggressive due to its early metastasis, rendering it one of the most lethal cancer types in the pediatric age group [2, 3]. Despite the rapid advancement of chemotherapy and surgery for osteosarcoma, the overall survival for patients with osteosarcoma has reached plateau in recent decades [4]; hence, it is of great urgency to learn the molecular and cellular mechanisms underlying the development of osteosarcomas, so as to reveal a novel target to treat the lethal disease.

A growing body of research has now shown that abnormal lipid metabolism is involved in the development of many types of cancer [5]. PLD, a ubiquitously expressed phospholipid-metabolizing enzyme that functions to catalyze the hydrolysis of phosphatidylcholine to generate phosphatidic acid and choline, is involved extensively in human cancers. The classic isoform of PLD, PLD1, is highly expressed in various human tumors, including in breast [6] and gastric cancer [7], and was stated to play a critical role in tumor progression [8]. Despite these advancements, little was known about its expression or biological significance in human osteosarcomas.

Sp1, which belongs to the Krüppel-like family of transcription factors, is a zinc finger transcription factor involved extensively in cell apoptosis and in growth, metabolism, and differentiation [9, 10]. In regard to cancer, numerous previous studies have shown that Sp1 levels were elevated in various types of human cancers, including lung [11], colorectal [12], gastric [13] and pancreatic ductal adenocarcinoma [14], correlating with aggressive biological activity and poor clinical outcomes [13, 15]. Mechanistically, elevated Sp1 levels lead to the expression of multiple oncogenic genes, causing progression of the cancers. Since PLD1 was also pro-tumoral in various cancers, we postulated that these factors were tightly correlated in osteosarcomas and function synergistically in promoting osteosarcoma initiation and progression.

In this study, we looked at the expression and biological significance of Sp1 and PLD1 in osteosarcomas. We found that both factors were elevated in this lethal disease. We also showed from a survival analysis and COX regression analyze that both factors have a prognostic value for osteosarcomas. Furthermore, our results showed the two factors were positively correlated, and patients with high expression of both exhibited the worst prognosis.

## RESULTS

### Clinicopathological characteristics of the patients

Among the osteosarcoma patients, 109 were male and 28 female, with ages ranging from 13 to 73 years old (mean age 20.3 years). The vast majority of tumors were located in the metaphysis of the long bones. Specifically, there were 85 lesions located in nearby knees, while were 42 in the humerus and the rest in other bones.

### Elevated PLD1 confers a poor prognosis for osteosarcoma

**Figure 1:**
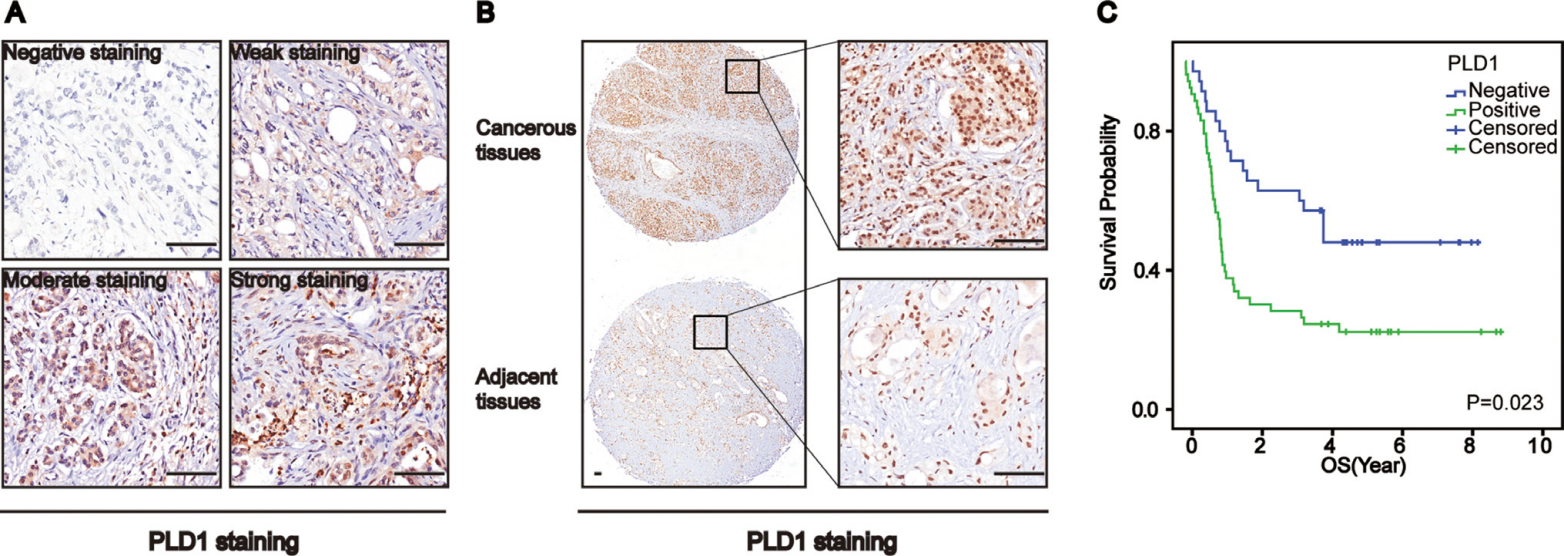
Phospholipase D1 (PLD1) confers a poor prognosis for patients with osteosarcomas (**A**) Representative images of PLD1 expression in osteosarcomas. (**B**) PLD1 expression in the cancerous tissue and paired non-cancerous tissue. (**C**) Overall survival curves based on PLD1 expression in osteosarcomas (patient with high expression vs low expression was 76: 61). Bar 200 um.

To learn the biological significance of PLD1 in osteosarcomas, we initially investigated its expression using immunohistochemistry. As shown in Figure 1A, PLD1 staining ranged from weak to strong in the patients. Statistically, among the 137 primary tumor samples, 68% showed positive staining, while about 32% showed negative staining. In addition, comparison of PLD1 expression between the cancerous tissue and adjacent normal tissue indicated that the cancerous tissue exhibited higher expression (*p* < 0.05, Table 1, Figure 1B). Additionally, the correlation analyze showed that PLD1 expression was positively correlated with the LDH (*p* = 0.041) and Eneeking stage (*p* = 0.000). Finally, a Kaplan–Meier survival analyzes indicated that PLD1 confers a poor prognosis (Figure 1C) and could be regarded as an independent prognostic factor for the patients (Table 2). Taken together, these data suggest that PLD1 was pro-tumoral in osteosarcomas, making it of great significance to reveal the mechanism whereby PLD1 facilitates osteosarcoma progression.

**Table 1:** The expression of PLD1 and Sp1 in the cancerous tissues and the adjacent tissues

	Number	PLD1 expression		*P* value	Sp1 expression		*P* value
		Positive	Negative		Positive	Negative	
**Osteosarcoma cancerous tissues**	137	76 (55.5%)	61 (44.5%)	*p* < 0.0001	73 (53.3%)	64 (46.7%)	*p* < 0.0001
**Osteosarcoma adjacent tissues**	137	19 (13.9%)	118 (86.1%)		27 (19.7%)	110 (80.3%)	

**Table 2:** Univariate and multivariate survival analysis of clinic-pathologic variables in osteosarcoma patients

Factor	Univariate analysis			Multivariate analysis		
	HR	95% CI	*P*	HR	95% CI	*P*
**Gender**						
Male vs. Female	0.61	0.181–2.053	0.425			
**Age**						
< 20 vs. > 20	0.44	0.174–1.117	0.804			
**Histologic grade**						
Well/moderately differentiated vs. Poorly differentiated	1.05	0.414–2.666	0.918			
**LDH (U/L)**						
< 600 vs. > 600	0.279	0.109–0.715	0.008*	0.324	0.126–0.836	0.020*
**Tumor size**						
< 6 cm vs. > 6 cm	5.61	2.421–13.000	0.000*	4.834	2.005–11.650	0.000*
**Primary tumor location**						
Femur, fibula and tibia vs. Humerus, scapula and rib	0.385	0.166–0.891	0.026*			
**Eneeking stage**						
≤ II vs. > II	3.985	1.743–9.112	0.001*			
Nuclear grade						
≤ II vs. > II	1.699	1.125–2.567	0.012*			
**PLD1**						
Positive vs. Negative	3.671	1.446–9.318	0.006*	3.087	1.204–7.914	0.019*
**Sp1**						
Positive vs. Negative	2.878	1.134–7.303	0.026*			
**Sp1/PLD1**						
Sp1+/PLD1+ vs. All others	3.522	1.543–8.042	0.003*	3.052	1.304–7.987	0.0023*

### Sp1 was elevated in osteosarcoma

**Figure 2:**
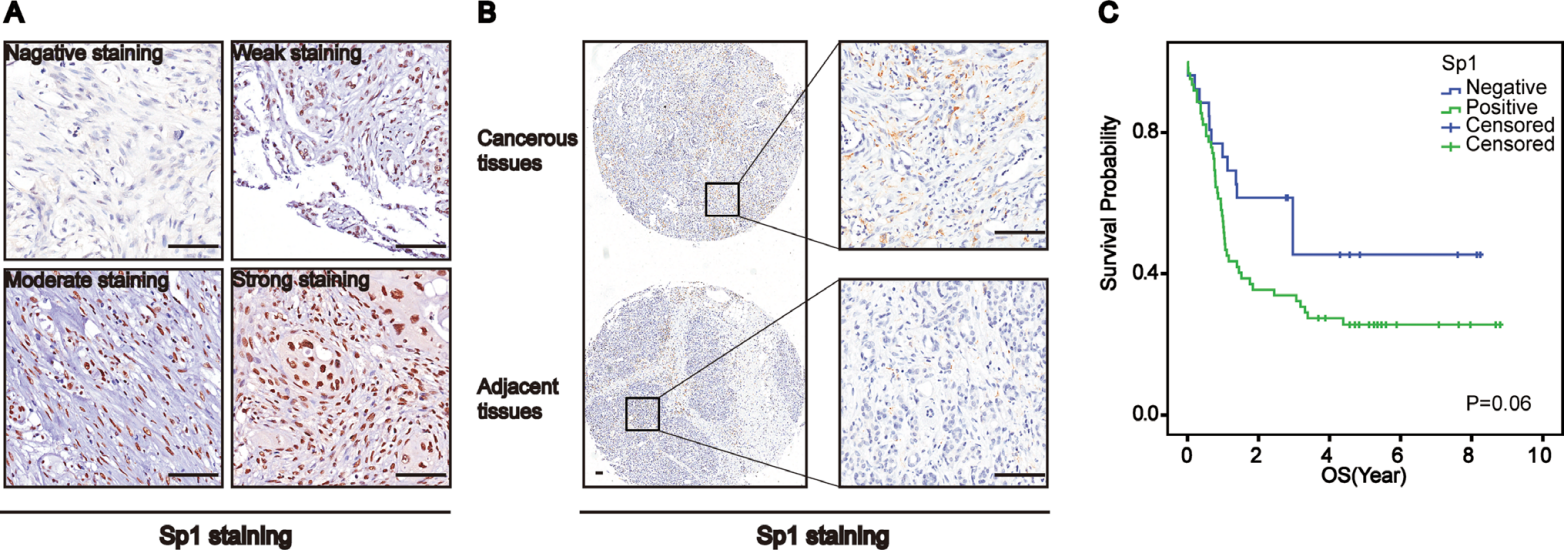
Specificity Protein 1 (Sp1) was upregulated in osteosarcomas (**A**) Representative images of Sp1 expression in osteosarcomas. (**B**) Sp1 expression in the cancerous tissue and paired non-cancerous tissue. (**C**) Overall survival curves based on Sp1 expression in osteosarcomas(patient with high expression vs low expression was 73: 64). Bar 200 um.

Prior studies have shown that the correlated Sp1 and PLD1 had a synergic effect in promoting pancreatic cancer, which indicates that Sp1 may also be elevated and have a pro-tumoral function in osteosarcomas. In order to test this, we investigated Sp1 expression in osteosarcomas using immunohistochemistry. Interestingly, we observed that Sp1 expression in osteosarcomas ranged from negative to strong (Figure 2A). Meanwhile, we also observed a significant difference in Sp1 expression between the cancerous tissue and adjacent normal tissue (Figure 2B, *p* < 0.05, Table 1). In addition, we found that Sp1 overexpression was correlated with the nuclear grade (*p* = 0.024), but not with other parameters of the patients. Unfortunately, the survival analysis showed no biological significance between Sp1 expression and the survival of patients (Figure 2C). These data indicate that Sp1 was not as important in osteosarcomas as expected.

### Synergic effect of Sp1 and PLD1 in promoting osteosarcoma progression

**Figure 3:**
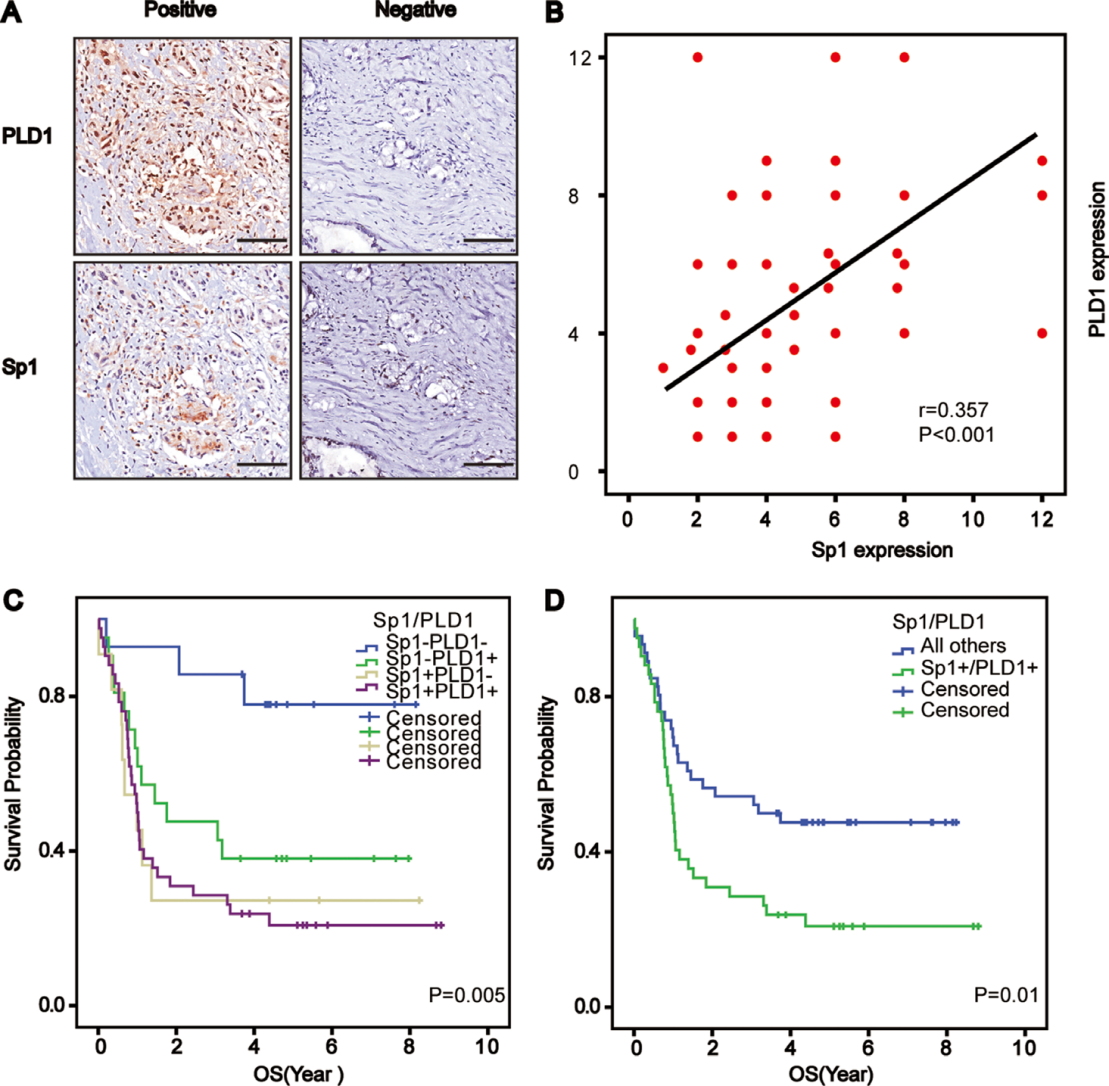
Correlated Sp1 and PLD1 confer the worst prognosis for osteosarcoma patients (**A**) Representative images depicting the positive correlation between PLD1 and Sp1 in the series sections of osteosarcomas. (**B**) Correlation analysis between PLD1 and Sp1 in osteosarcomas. (**C**, **D**) Overall survival curves based on PLD1, Sp1 and their combined expression in osteosarcoma patients (patient with co-high expression of Sp1 and PLD1 vs other patient low expression was 37: 100). Bar 200 um.

Considering the biological significance of PLD1 in osteosarcomas, and the fact that Sp1 could transcriptionally activate genes associating with malignant phenotypes, we postulated that the two factors were positively correlated. To test this theory, we detected Sp1 and PLD1 expression under the microscope, which were found in serial sections of the tissue array where PLD1 staining was accompanied by Sp1 staining, and vice versa (Figure 3A). A Spearman’s rank test was conducted on the data, showing that PLD1 was positively correlated with Sp1 in the patients (*r* = 0.357; *p* < 0.001, Figure 3B). More importantly, the survival analysis indicated that patients with both PLD1 and Sp1 over-expression tended to have the worst survival among all the patients (Figure 3C, 3D), and that their co-high expression could be treated as an independent prognostic factor for the patients (*p* = 0.0023; HR = 3.052; 95% CI 1.304–7.987). Additionally, the Cox regression model showed that LDH expression (*p* = 0.020; HR = 0.324; 95% CI 0.126–0.836) and tumor size (*p* = 0.000; HR = 4.834; 95% CI 2.005–11.650) were also independent prognostic factors for the patients (Table 2).

## DISCUSSION

Abnormal metabolism was suggested to play a critical role in oncogenesis, tumor progression, and metastasis [16]. In this study, we examined the expression and prognostic significance of PLD1 - a critical enzyme involved in lipid metabolism - in osteosarcomas. Our results showed that PLD1 was elevated in the disease associated with a poor prognosis. We also revealed that PLD1 was positively correlated with Sp1- a factor that was also elevated in osteosarcomas. Moreover, we showed that co-high expression of both PLD1 and Sp1 conferred the worst prognosis for patients, with their high expression acting as an independent prognostic factor for the patients. Taken together, our data revealed that therapeutic inhibition of PLD1, either without or in combination with Sp1, could provide an alternative approach for the management of osteosarcoma.

Elevated PLD1 has been reported in various malignant tumors responsible for angiogenesis, metastasis, and invasion, via multiple signal pathways [17, 18]. For instance, Chen and colleagues found that angiogenesis and metastasis were decreased in mice with decreased PLD1 expression [17]. It has also been reported that platelet derived growth factor (PDGF) increased PLD1, which could then facilitate the invasion of breast cancer cells [19]. Despite this, no studies have systematically examined the expression pattern and prognostic value of PLD1 in osteosarcomas. To our best knowledge, our findings provide the first evidence that PLD1 is pro-tumoral in osteosarcoma and that targeted inhibition of PLD1 could represent a novel approach to treatment of the disease.

Sp1 is a C2H2-type zinc finger-type transcription factor which binds the GC-rich sequences of target genes. As stated, it was upregulated in various malignant tumor cells associated with the malignant phenotype; for example, Sp1 was elevated in advanced nasopharyngeal carcinoma responsible for proliferation as well as clonogenicity [20]. In addition, Hang et al. reported that Sp1 could upregulate COX-2 expression, which was positively associated with lymph node metastasis in pancreatic ductal adenocarcinoma (PDAC), predicting a poor prognosis for patients [21]. We have consistently shown that Sp1 was elevated in osteosarcomas and was associated with the nuclear grade (*p* = 0.024), but not with the survival of the patients. These data might reveal that the role of Sp1 in human malignancies was tissue-specific in this case. Also, this could attribute to the less sample size of our study.

Since Sp1 is transcription factor and transcription activation is the primary machinery linking it to other factors. In a prior study, Park et al. revealed that Sp1 was an essential transcription factor linking PLD1 to matrix metalloproteinases-2 (MMP-2), which contributed to the invasion of the glioma [22]. At the same time, another study found that elevated Sp1 levels increased PLD1 promoter activity in drosophila SL2 cells [23]. Since our study found a positive correlation between Sp1 and PLD1, we postulate here that transcription activation might also apply to the positive correlation between Sp1 and PLD1 in osteosarcomas.

Taken together, our study finds that PLD1 was elevated in osteosarcomas, and that its high expression was an independent prognostic factor for the disease, suggesting that PLD1 can serve as a pro-tumoral factor in osteosarcomas. Since PLD1, which was also associated with the malignant phenotype of osteosarcomas, was positively correlated with Sp1, further studies are required to determine the potential role of targeted inhibition of PLD1 without/or in combination with Sp1 as an candidate therapeutic target in clinics.

## MATERIALS AND METHODS

### Patients

A total of 137 patients with a histopathological diagnosis of osteosarcoma were enrolled in this study at the Second Affiliate Hospital of the University of South China (Hengyang, China), between 2010 and 2014. Paraffin-embedded cancer specimens for each patient were obtained from the pathology department of the Second Affiliate Hospital of the University of South China. The last follow-up visit was on September 28th, 2016. Histological types were defined according to the WHO classification and tumor stage was classified using the Eneeking staging system [24]. The baseline characteristics of patients collected included age, gender, tumor size, primary tumor location, nerve invasion, vascular invasion, and nuclear grade (Table 3). The hospital ethics committee approved the study and each patient provided written, informed consent.

**Table 3:** Relationship between expression of Sp1, PLD1 and clinicopathlogical parameters in osteosarcoma

Factor	Number	Sp1		*P*	PLD1		*P*
		Negative	Positive		Negative	Positive	
**Gender**							
**Male**	109	54 (39.4%)	55 (40.1%)	0.191	48 (35.0%)	61 (44.6%)	0.82
**Female**	28	10 (7.3%)	18 (13.1%)		13 (9.5%)	15 (11.0%)	
**Age**							
**≥ 20**	75	37 (27.0%)	38 (27.7%)	0.606	34 (24.8%)	41 (29.9%)	0.864
**< 20**	62	27 (19.7%)	35 (25.5%)		27 (19.7%)	35 (25.5%)	
**Histologic grade**							
**Well**	22	10 (7.3%)	12 (8.8%)	0.178	7 (5.1%)	15 (11.0%)	0.418
**Moderately**	80	33 (24.1%)	47 (34.3%)		38 (27.7%)	42 (30.7%)	
**Poorly**	35	21 (15.3%)	14 (10.2%)		16 (11.7%)	19 (13.9%)	
**LDH (U/L)**							
**< 300**	63	36 (26.3%)	27 (19.7%)	0.041*	29	34	0.783
**300–600**	71	28 (20.4%)	43 (31.4%)		31	40	
**> 600**	3	0	3 (2.2%)		1	2	
**Tumor size**							
**< 3 cm**	23	10 (7.3%)	13 (9.5%)	0.187	11 (8.0%)	12 (8.8%)	0.524
**3–6 cm**	79	33 (24.1%)	46 (33.6%)		32 (23.4%)	47 (34.3%)	
**> 6 cm**	35	21 (15.3%)	14 (10.2%)		18 (13.1%)	17 (12.4%)	
**Tumor location**							
**Humerus, scapula**	49	25 (18.2%)	24 (17.5%)	0.566	21 (15.3%)	28 (20.4%)	0.734
**Femur, fibula and tibia**	85	39 (28.5%)	46 (33.6%)		39 (28.5%)	46 (33.6%)	
**Rib**	3	0	3 (2.2%)		1 (0.7%)	2 (1.5%)	
**Eneeking stage**							
**I**	48	11 (8.0%)	37 (27.0%)	0.000*	16 (11.7%)	32 (23.4%)	0.059
**II**	49	31 (22.6%)	18 (13.1%)		28 (20.4%)	21 (15.3%)	
**III**	40	22 (16.1%)	18 (13.1%)		17 (12.4%)	23 (16.8%)	
**Nuclear grade**							
**I**	104	48 (35.0%)	56 (40.9%)	0.717	42 (30.7%)	62 (45.3%)	0.024*
**II**	28	14 (10.2%)	14 (10.2%)		18 (13.1%)	10 (7.3%)	
**III**	5	2 (1.5%)	3 (2.2%)		1 (0.7%)	4 (3.0%)	

### Tissue microarray construction

In brief, core samples were obtained from representative regions of each tumor based on hematoxylin and eosin staining. Representative tumor regions were defined as areas that were at least 75% cancerous cells without necrosis. Tissue cylinders (1.5 mm in diameter) were then punched from these regions of the block using a tissue microarrayer (Gentury, IL, USA) and placed into recipient paraffin blocks. Two sets of three paraffin-embedded tissue microarray (TMA) blocks were made. Sections of the resulting TMA blocks were transferred to glass slides. In total, two sets of TMAs, which contained 137 tumor tissue spots, were used in this study.

### Immunohistochemistry

Paraffin tissue sections were de-waxed in xylene and re-hydrated via graded alcohols. Endogenous peroxidase activity was blocked with 0.3% hydrogen peroxide for 10 mins, before antigen retrieval was undertaken by setting the slides in a 0.01 M citrate buffer (pH 6.0) at 98°C for 5 min using a microwave oven. The slides were then cooled to room temperature and blocked by incubating them with normal goat serum at room temperature for 1 h, followed by incubation at 4°C overnight with the primary antibodies (Cell Signaling Technology, Beverly, MA, USA). Finally, the sections were incubated with the HRP-labeled secondary antibody and visualized using diaminobenzidine.

### Evaluation of PLD1 and Sp1 staining

Evaluation of the staining in at least five areas was performed at 400 × magnification by two independent pathologists blind to the clinical information about the patients. The staining was scored according to the intensity and percentage of stained cells. Staining intensity was assigned to be 0 (no staining), 1 (weak staining), 2 (moderate staining), or 3 (strong staining). The staining percentage was graded as follows: 0, < 10%; 1, 10–30%; 2, 31–50%; 3, 51–75%; 4, > 75%. The final scores were calculated from the staining intensity × the percentage of positive cells. For statistical analyses, a score < 6 was regarded as negative, and > 6 as highly positive.

### Statistical analysis

Statistical analyses were performed using SPSS software (version 21.0; SPSS Inc., Chicago, IL, USA). Associations between Sp1 and PLD1 expression levels and potential prognostic factors were analyzed using the χ^2^ test, while The Spearman’s rank test was used to evaluate their correlation. The Kaplan–Meier survival estimates and log-rank tests were used to evaluate survival data based on Sp1 and PLD1 expression. The Cox proportional hazards model was used for multivariate analysis of prognostic factors. A value of *p* < 0.05 was considered as statistically significant.

## References

[1] Luetke A, Meyers PA, Lewis I, Juergens H (2014). Osteosarcoma treatment–where do we stand? A state of the art review. Cancer treatment reviews.

[2] Fiori R, Vivo DD, Scarano AL, D'Onofrio S, Calabria E, Giovanni S (2015). Local invasion of jaw osteosarcoma. International Journal of Case Reports and Images (IJCRI).

[3] Chou AJ, Kleinerman ES, Krailo MD, Chen Z, Betcher DL, Healey JH, Conrad EU, Nieder ML, Weiner MA, Wells RJ, Womer RB, Meyers PA (2009). Addition of muramyl tripeptide to chemotherapy for patients with newly diagnosed metastatic osteosarcoma: a report from the Children's Oncology Group. Cancer.

[4] Marina N, Gebhardt M, Teot L, Gorlick R (2004). Biology and therapeutic advances for pediatric osteosarcoma. Oncologist.

[5] Santos CR, Schulze A (2012). Lipid metabolism in cancer. FEBS Journal.

[6] Noh DY, Ahn SJ, Lee RA, Park IA, Kim JH, Suh PG, Ryu SH, Lee KH, Han JS (2000). Overexpression of phospholipase D1 in human breast cancer tissues. Cancer letters.

[7] Kang D, Hwang W, Park M, Ko G, Ha W, Kim K, Lee Y, Choi K, Min D (2013). Rebamipide abolishes Helicobacter pylori CagA-induced phospholipase D1 expression via inhibition of NFκB and suppresses invasion of gastric cancer cells. Oncogene.

[8] Park MH (2011). Quercetin-induced downregulation of phospholipase D1 inhibits proliferation and invasion in U87 glioma cells. Biochemical and biophysical research communications.

[9] Marin M, Karis A, Visser P, Grosveld F, Philipsen S (1997). Transcription factor Sp1 is essential for early embryonic development but dispensable for cell growth and differentiation. Cell.

[10] Zhu J, Sun Y, Luo J, Wu M, Li J, Cao Y (2015). Specificity protein 1 regulates gene expression related to fatty acid metabolism in goat mammary epithelial cells. Int J Mol Sci.

[11] Kong LM, Liao CG, Fei F, Guo X, Xing JL, Chen ZN (2010). Transcription factor Sp1 regulates expression of cancer-associated molecule CD147 in human lung cancer. Cancer science.

[12] Wang F, Ma YL, Zhang P, Shen TY, Shi CZ, Yang YZ, Moyer MP, Zhang HZ, Chen HQ, Liang Y (2013). SP1 mediates the link between methylation of the tumour suppressor miR-149 and outcome in colorectal cancer. J Pathol.

[13] Wang L, Wei D, Huang S, Peng Z, Le X, Wu TT, Yao J, Ajani J, Xie K (2003). Transcription factor Sp1 expression is a significant predictor of survival in human gastric cancer. Clinical Cancer Research.

[14] Jiang NY, Woda BA, Banner BF, Whalen GF, Dresser KA, Lu D (2008). Sp1, a new biomarker that identifies a subset of aggressive pancreatic ductal adenocarcinoma. Cancer Epidemiology Biomarkers & Prevention.

[15] Hsu TI, Wang M, Chen S, Yeh Y, Su W, Chang WC, Hung J (2012). Sp1 expression regulates lung tumor progression. Oncogene.

[16] Sahar S, Sassone-Corsi P (2009). Metabolism and cancer: the circadian clock connection. Nature Reviews Cancer.

[17] Chen Q, Sato T, Hongu T, Zhang Y, Ali W, Cavallo JA, van der Velden A, Tian H, Di Paolo G, Nieswandt B (2012). Key roles for the lipid signaling enzyme phospholipase d1 in the tumor microenvironment during tumor angiogenesis and metastasis. Science signaling.

[18] Kang DW, Park MH, Lee YJ, Kim HS, Lindsley CW, Alex Brown H, Min do S (2011). Autoregulation of phospholipase D activity is coupled to selective induction of phospholipase D1 expression to promote invasion of breast cancer cells. International Journal of Cancer.

[19] Kang DW (2010). Platelet derived growth factor increases phospholipase D1 but not phospholipase D2 expression via NFκB signaling pathway and enhances invasion of breast cancer cells. Cancer letters.

[20] Zhang JP, Zhang H, Wang HB, Li YX, Liu GH, Xing S, Li MZ, Zeng MS (2014). Down-regulation of Sp1 suppresses cell proliferation, clonogenicity and the expressions of stem cell markers in nasopharyngeal carcinoma. Journal of translational medicine.

[21] Hang J, Hu H, Huang J, Han T, Zhuo M, Zhou Y, Wang L, Wang Y, Jiao F (2016). Sp1 and COX2 expression is positively correlated with a poor prognosis in pancreatic ductal adenocarcinoma. Oncotarget.

[22] Park MH, Ahn BH, Hong YK (2009). Overexpression of phospholipase D enhances matrix metalloproteinase-2 expression and glioma cell invasion via protein kinase C and protein kinase A/NF-κB/Sp1-mediated signaling pathways. Carcinogenesis.

[23] Gao S, Murakami M, Ito H, Furuhata A, Yoshida K, Tagawa Y, Hagiwara K, Takagi A, Kojima T, Suzuki M (2009). Mutated RAS Induced PLD1 Gene Expression through Increased Sp1 Trascription Factor. Nagoya journal of medical science.

[24] Enneking WF, Spanier SS, Goodman MA (1980). A system for the surgical staging of musculoskeletal sarcoma. Clinical orthopaedics and related research.

